# (Cyano­meth­yl)triphenyl­phospho­nium chloride

**DOI:** 10.1107/S1600536808034673

**Published:** 2008-10-31

**Authors:** Muhammad Shafiq, M. Nawaz Tahir, Islam Ullah Khan, Muhammad Nadeem Arshad, Zaib-un-Nisa

**Affiliations:** a Government College University, Department of Chemistry, Lahore, Pakistan; b University of Sargodha, Department of Physics, Sagrodha, Pakistan

## Abstract

In the mol­ecule of the title compound, C_20_H_17_NP^+^·Cl^−^, the coordination around the P atom is slightly distorted tetra­hedral. In the crystal structure, inter­molecular C—H⋯N and C—H⋯Cl hydrogen bonds link the mol­ecules. There is a π–π contact between the phenyl rings [centroid–centroid distance = 3.702 (3) Å].

## Related literature

For related structures, see: Czerwinski (2004 ▶); Czerwinski & Ponnuswamy (1988 ▶); de Dubourg *et al.* (1986 ▶); Fischer & Wiebelhaus (1997 ▶); Shafiq *et al.* (2008 ▶); Skapski & Stephens (1974 ▶); Tahir *et al.* (2008 ▶).
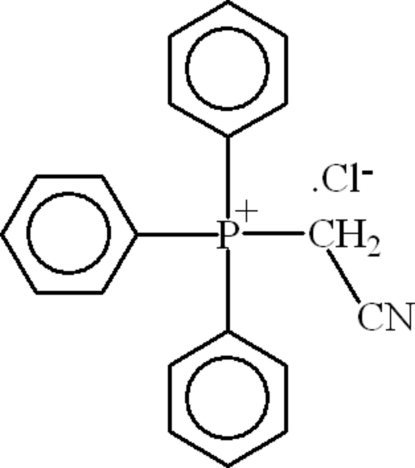


## Experimental

### 

#### Crystal data

C_20_H_17_NP^+^·Cl^−^
*M* *_r_* = 337.77
Monoclinic, 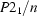

*a* = 11.8269 (5) Å
*b* = 11.8130 (4) Å
*c* = 12.8918 (5) Å
β = 92.213 (2)°
*V* = 1799.79 (12) Å^3^
*Z* = 4
Mo *K*α radiation
μ = 0.30 mm^−1^
*T* = 296 (2) K
0.26 × 0.20 × 0.16 mm

#### Data collection

Bruker Kappa APEXII CCD diffractometer
Absorption correction: multi-scan (*SADABS*; Bruker, 2005 ▶) *T*_min_ = 0.928, *T*_max_ = 0.950
19927 measured reflections
4465 independent reflections
3145 reflections with *I* > 2σ(*I*)
*R*_int_ = 0.034

#### Refinement

*R*[*F* ^2^ > 2σ(*F* ^2^)] = 0.041
*wR*(*F* ^2^) = 0.111
*S* = 1.03
4465 reflections
208 parameters
H-atom parameters constrained
Δρ_max_ = 0.37 e Å^−3^
Δρ_min_ = −0.31 e Å^−3^

### 

Data collection: *APEX2* (Bruker, 2007 ▶); cell refinement: *SAINT* (Bruker, 2007 ▶); data reduction: *SAINT*; program(s) used to solve structure: *SHELXS97* (Sheldrick, 2008 ▶); program(s) used to refine structure: *SHELXL97* (Sheldrick, 2008 ▶); molecular graphics: *ORTEP-3 for Windows* (Farrugia, 1997 ▶) and *PLATON* (Spek, 2003 ▶); software used to prepare material for publication: *WinGX* (Farrugia, 1999 ▶) and *PLATON*.

## Supplementary Material

Crystal structure: contains datablocks global, I. DOI: 10.1107/S1600536808034673/hk2558sup1.cif

Structure factors: contains datablocks I. DOI: 10.1107/S1600536808034673/hk2558Isup2.hkl

Additional supplementary materials:  crystallographic information; 3D view; checkCIF report

## Figures and Tables

**Table:** 

P1—C1	1.7923 (18)
P1—C7	1.7845 (18)
P1—C13	1.7851 (17)
P1—C19	1.8046 (17)

**Table:** 

C1—P1—C7	111.03 (8)
C1—P1—C13	109.26 (8)
C1—P1—C19	108.56 (8)
C7—P1—C13	110.71 (8)
C7—P1—C19	106.81 (8)
C13—P1—C19	110.43 (8)

**Table 2:** Hydrogen-bond geometry (Å, °)

*D*—H⋯*A*	*D*—H	H⋯*A*	*D*⋯*A*	*D*—H⋯*A*
C12—H12⋯Cl1	0.93	2.66	3.479 (2)	147
C17—H17⋯N1^i^	0.93	2.61	3.530 (3)	171
C19—H19*A*⋯Cl1^ii^	0.97	2.34	3.3076 (17)	173
C19—H19*B*⋯Cl1^iii^	0.97	2.46	3.3830 (19)	160

Symmetry codes: (i) 
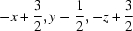
; (ii) 
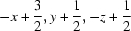
; (iii) 
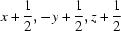
; (iv) 
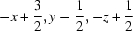
.

**Fig. 1.**
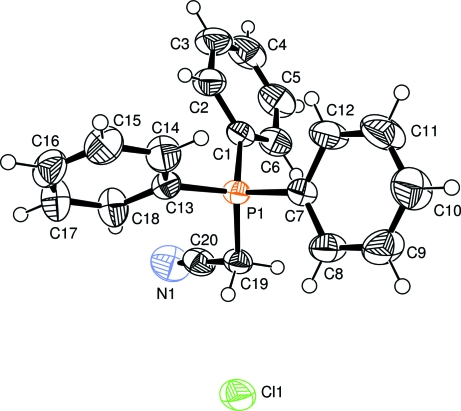
The molecular structure of the title molecule, with the atom-numbering scheme. Displacement ellipsoids drawn at the 50% probability level.

**Fig. 2.**
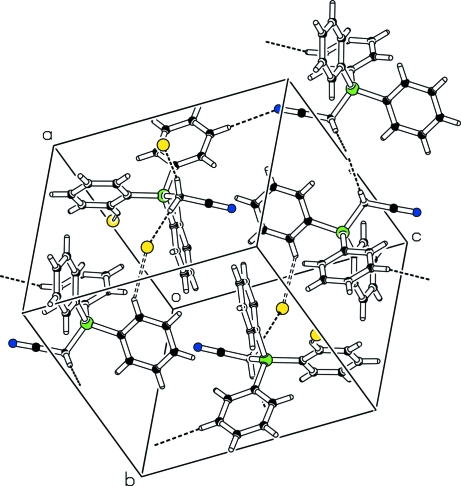
A partial packing diagram. Hydrogen bonds are shown as dashed lines.

## supplementary crystallographic information

### Comment

Triphenyl phosphonium compounds are key reagents in the Wittig reactions, used to convert aldehydes and ketones into alkenes. The Wittig reaction has seen use in applications ranging from the synthesis of simple alkenes to the construction of complex biologically active molecules for the pharmaceutical industry.The title compound is synthesized for the derivatization of our already published structures (Shafiq *et al.*,  2008; Tahir *et al.*,  2008) using this particular reaction. Various structures have been published having the similar geometry around P atom (Skapski & Stephens, 1974; de Dubourg *et al.*, 1986; Czerwinski & Ponnuswamy, 1988; Fischer & Wiebelhaus, 1997; Czerwinski, 2004).

In the molecule of the title compound (Fig 1), the geometry around P atom is slightly distorted tetrahedral (Table 1). Rings A (C1-C6), B (C7-C12) and C (C13-C18) are of course planar. The dihedral angles between them are A/B = 86.10 (11)°, A/C = 89.78 (10)° and B/C = 76.23 (12)°.

In the crystal structure, intramolecular C-H···Cl and intermolecular C-H···N and C-H···Cl hydrogen bonds (Table 2) link the molecules (Fig. 2), in which they may be effective in the stabilization of the structure. The π—π contact between the phenyl rings, Cg3···Cg3^i^ [symmetry code: (i) 2 - x, -y, 1 - z, where Cg3 is the centroid of the ring C (C13-C18)] may further stabilize the structure, with centroid-centroid distance of 3.702 (3) Å. There also exist a C—H···π contact (Table 2) between the phenyl rings.

### Experimental

Triphenylphosphine (10 g, 0.038 mol) was dissolved in benzene (20 ml) under stirring at room temperature. To this solution, chloroacetonitrile (4 g, 0.0514 mole) was added dropwise. After complete addition, clear solution formed was left in the darkness for 2-3 d. Colorless crystals formed were separated for X-ray diffraction studies.

### Refinement

H-atoms were positioned geometrically, with C-H = 0.93 and 0.97 Å for aromatic and methylene H, and constrained to ride on their parent atoms, with U_iso_(H) = 1.2U_eq_(C).

### Crystal data

**Table:**

C_20_H_17_NP^+^·Cl^−^	*F*(000) = 704
*M**_r_* = 337.77	*D*_x_ = 1.247 Mg m^−^^3^
Monoclinic, *P*2_1_/*n*	Mo *K*α radiation, λ = 0.71073 Å
Hall symbol: -P 2yn	Cell parameters from 4467 reflections
*a* = 11.8269 (5) Å	θ = 2.3–28.3°
*b* = 11.8130 (4) Å	µ = 0.30 mm^−^^1^
*c* = 12.8918 (5) Å	*T* = 296 K
β = 92.213 (2)°	Prismatic, colorless
*V* = 1799.79 (12) Å^3^	0.26 × 0.20 × 0.16 mm
*Z* = 4	

### Data collection

**Table:**

Bruker KappaAPEXII CCD diffractometer	4465 independent reflections
Radiation source: fine-focus sealed tube	3145 reflections with *I* > 2σ(*I*)
graphite	*R*_int_ = 0.034
Detector resolution: 7.40 pixels mm^-1^	θ_max_ = 28.3°, θ_min_ = 2.3°
ω scans	*h* = −15→15
Absorption correction: multi-scan (SADABS; Bruker, 2005)	*k* = −10→15
*T*_min_ = 0.928, *T*_max_ = 0.950	*l* = −17→14
19927 measured reflections	

### Refinement

**Table:**

Refinement on *F*^2^	Primary atom site location: structure-invariant direct methods
Least-squares matrix: full	Secondary atom site location: difference Fourier map
*R*[*F*^2^ > 2σ(*F*^2^)] = 0.041	Hydrogen site location: inferred from neighbouring sites
*wR*(*F*^2^) = 0.111	H-atom parameters constrained
*S* = 1.03	*w* = 1/[σ^2^(*F*_o_^2^) + (0.0464*P*)^2^ + 0.4839*P*] where *P* = (*F*_o_^2^ + 2*F*_c_^2^)/3
4465 reflections	(Δ/σ)_max_ < 0.001
208 parameters	Δρ_max_ = 0.37 e Å^−^^3^
0 restraints	Δρ_min_ = −0.31 e Å^−^^3^

### Special details

**Table:**

Geometry. Bond distances, angles *etc*. have been calculated using the rounded fractional coordinates. All su's are estimated from the variances of the (full) variance-covariance matrix. The cell e.s.d.'s are taken into account in the estimation of distances, angles and torsion angles
Refinement. Refinement of *F*^2^ against ALL reflections. The weighted *R*-factor *wR* and goodness of fit *S* are based on *F*^2^, conventional *R*-factors *R* are based on *F*, with *F* set to zero for negative *F*^2^. The threshold expression of *F*^2^ > σ(*F*^2^) is used only for calculating *R*-factors(gt) *etc*. and is not relevant to the choice of reflections for refinement. *R*-factors based on *F*^2^ are statistically about twice as large as those based on *F*, and *R*- factors based on ALL data will be even larger.

### Fractional atomic coordinates and isotropic or equivalent isotropic displacement parameters (Å^2^)

**Table:**

	*x*	*y*	*z*	*U*_iso_*/*U*_eq_	
Cl1	0.64049 (5)	0.13296 (4)	0.08789 (5)	0.0652 (2)	
P1	0.81585 (4)	0.28430 (4)	0.41401 (3)	0.0347 (1)	
N1	0.7328 (2)	0.38278 (18)	0.67432 (16)	0.0773 (8)	
C1	0.66854 (15)	0.30928 (15)	0.38497 (13)	0.0396 (5)	
C2	0.59647 (17)	0.22052 (19)	0.35858 (15)	0.0539 (7)	
C3	0.48330 (19)	0.2418 (3)	0.33767 (18)	0.0708 (9)	
C4	0.4421 (2)	0.3502 (3)	0.34392 (19)	0.0758 (9)	
C5	0.51305 (19)	0.4389 (2)	0.36860 (17)	0.0674 (8)	
C6	0.62701 (17)	0.41952 (18)	0.38895 (15)	0.0530 (7)	
C7	0.89873 (15)	0.31735 (14)	0.30527 (13)	0.0392 (5)	
C8	1.00699 (17)	0.36037 (18)	0.31824 (15)	0.0537 (7)	
C9	1.06821 (19)	0.3851 (2)	0.23237 (18)	0.0710 (9)	
C10	1.0223 (2)	0.3654 (3)	0.13446 (19)	0.0809 (10)	
C11	0.9166 (2)	0.3196 (3)	0.12143 (17)	0.0825 (12)	
C12	0.85329 (18)	0.2966 (2)	0.20620 (15)	0.0620 (8)	
C13	0.83557 (14)	0.14007 (14)	0.45185 (14)	0.0386 (5)	
C14	0.86489 (19)	0.05919 (17)	0.37959 (17)	0.0576 (7)	
C15	0.8752 (2)	−0.05313 (17)	0.40849 (19)	0.0640 (8)	
C16	0.85644 (18)	−0.08481 (17)	0.50791 (19)	0.0611 (8)	
C17	0.8289 (2)	−0.00608 (19)	0.58004 (18)	0.0663 (8)	
C18	0.81820 (19)	0.10688 (17)	0.55274 (16)	0.0552 (7)	
C19	0.86206 (15)	0.37784 (14)	0.51789 (13)	0.0404 (5)	
C20	0.78895 (19)	0.37952 (16)	0.60511 (16)	0.0501 (7)	
H2	0.62426	0.14704	0.35500	0.0647*	
H3	0.43471	0.18264	0.31927	0.0849*	
H4	0.36530	0.36362	0.33129	0.0908*	
H5	0.48444	0.51206	0.37164	0.0809*	
H6	0.67549	0.47949	0.40513	0.0636*	
H8	1.03834	0.37259	0.38456	0.0644*	
H9	1.14070	0.41504	0.24066	0.0853*	
H10	1.06347	0.38349	0.07668	0.0970*	
H11	0.88740	0.30382	0.05498	0.0990*	
H12	0.78055	0.26742	0.19721	0.0744*	
H14	0.87764	0.08059	0.31161	0.0690*	
H15	0.89494	−0.10715	0.35994	0.0767*	
H16	0.86246	−0.16063	0.52674	0.0733*	
H17	0.81722	−0.02839	0.64798	0.0795*	
H18	0.79937	0.16027	0.60220	0.0662*	
H19A	0.86739	0.45405	0.49048	0.0485*	
H19B	0.93730	0.35522	0.54233	0.0485*	

### Atomic displacement parameters (Å^2^)

**Table:**

	*U*^11^	*U*^22^	*U*^33^	*U*^12^	*U*^13^	*U*^23^
Cl1	0.0647 (3)	0.0431 (3)	0.0854 (4)	−0.0019 (2)	−0.0289 (3)	−0.0085 (2)
P1	0.0349 (2)	0.0353 (2)	0.0335 (2)	0.0008 (2)	−0.0029 (2)	−0.0028 (2)
N1	0.0872 (15)	0.0903 (16)	0.0549 (12)	0.0070 (12)	0.0102 (11)	−0.0203 (11)
C1	0.0354 (9)	0.0521 (10)	0.0312 (9)	0.0023 (8)	−0.0011 (7)	−0.0010 (7)
C2	0.0478 (11)	0.0621 (12)	0.0512 (12)	−0.0067 (10)	−0.0057 (9)	−0.0032 (10)
C3	0.0468 (13)	0.0974 (19)	0.0669 (15)	−0.0187 (12)	−0.0128 (11)	0.0019 (13)
C4	0.0408 (12)	0.122 (2)	0.0638 (15)	0.0114 (14)	−0.0064 (10)	0.0088 (15)
C5	0.0521 (13)	0.0833 (16)	0.0662 (14)	0.0248 (12)	−0.0052 (11)	−0.0011 (12)
C6	0.0466 (11)	0.0597 (12)	0.0522 (12)	0.0102 (9)	−0.0052 (9)	−0.0023 (10)
C7	0.0379 (9)	0.0427 (9)	0.0367 (9)	0.0010 (7)	−0.0009 (7)	−0.0030 (7)
C8	0.0439 (11)	0.0720 (14)	0.0451 (11)	−0.0083 (10)	0.0007 (9)	−0.0115 (10)
C9	0.0478 (12)	0.103 (2)	0.0630 (15)	−0.0196 (12)	0.0138 (11)	−0.0119 (13)
C10	0.0630 (16)	0.129 (2)	0.0521 (14)	−0.0080 (15)	0.0210 (12)	0.0016 (14)
C11	0.0623 (16)	0.148 (3)	0.0372 (12)	−0.0081 (16)	0.0014 (10)	−0.0048 (14)
C12	0.0448 (11)	0.1007 (18)	0.0402 (11)	−0.0116 (11)	−0.0036 (9)	−0.0066 (11)
C13	0.0354 (9)	0.0345 (8)	0.0458 (10)	0.0013 (7)	−0.0001 (7)	−0.0017 (7)
C14	0.0790 (15)	0.0447 (11)	0.0492 (12)	0.0012 (10)	0.0054 (10)	−0.0090 (9)
C15	0.0814 (16)	0.0399 (10)	0.0704 (15)	0.0028 (10)	0.0010 (12)	−0.0138 (10)
C16	0.0608 (13)	0.0360 (10)	0.0862 (17)	0.0014 (9)	−0.0024 (12)	0.0041 (10)
C17	0.0838 (16)	0.0533 (12)	0.0625 (14)	0.0078 (12)	0.0131 (12)	0.0171 (11)
C18	0.0703 (14)	0.0457 (10)	0.0505 (12)	0.0111 (10)	0.0143 (10)	0.0026 (9)
C19	0.0452 (10)	0.0372 (9)	0.0382 (9)	0.0018 (7)	−0.0078 (8)	−0.0047 (7)
C20	0.0595 (12)	0.0485 (11)	0.0418 (11)	0.0057 (9)	−0.0060 (10)	−0.0116 (8)

### Geometric parameters (Å, °)

**Table:**

P1—C1	1.7923 (18)	C16—C17	1.364 (3)
P1—C7	1.7845 (18)	C17—C18	1.385 (3)
P1—C13	1.7851 (17)	C19—C20	1.445 (3)
P1—C19	1.8046 (17)	C2—H2	0.9300
N1—C20	1.133 (3)	C3—H3	0.9300
C1—C2	1.385 (3)	C4—H4	0.9300
C1—C6	1.394 (3)	C5—H5	0.9300
C2—C3	1.378 (3)	C6—H6	0.9300
C3—C4	1.374 (5)	C8—H8	0.9300
C4—C5	1.372 (4)	C9—H9	0.9300
C5—C6	1.382 (3)	C10—H10	0.9300
C7—C8	1.382 (3)	C11—H11	0.9300
C7—C12	1.388 (3)	C12—H12	0.9300
C8—C9	1.377 (3)	C14—H14	0.9300
C9—C10	1.375 (3)	C15—H15	0.9300
C10—C11	1.367 (4)	C16—H16	0.9300
C11—C12	1.376 (3)	C17—H17	0.9300
C13—C14	1.388 (3)	C18—H18	0.9300
C13—C18	1.381 (3)	C19—H19A	0.9700
C14—C15	1.382 (3)	C19—H19B	0.9700
C15—C16	1.362 (3)		
			
Cl1···C19^i^	3.3076 (17)	C14···H2	3.0300
Cl1···C16^ii^	3.556 (2)	C15···H12^i^	3.0900
Cl1···C19^iii^	3.3830 (19)	C18···H10^viii^	3.0400
Cl1···C12	3.479 (2)	C19···H18	2.9000
Cl1···H16^ii^	2.8500	C19···H8	2.7500
Cl1···H19A^i^	2.3400	C19···H6	2.8600
Cl1···H8^iii^	2.8400	C20···H6	3.0900
Cl1···H19B^iii^	2.4600	C20···H18	2.5900
Cl1···H12	2.6600	H2···C14	3.0300
Cl1···H6^i^	2.8300	H2···C13	2.7500
N1···H18	2.9100	H5···N1^v^	2.8900
N1···H17^iv^	2.6100	H6···Cl1^ii^	2.8300
N1···H5^v^	2.8900	H6···H19A	2.5000
C6···C20	3.353 (3)	H6···C19	2.8600
C12···C14	3.586 (3)	H6···C20	3.0900
C12···Cl1	3.479 (2)	H8···H19B	2.4100
C12···C15^ii^	3.512 (3)	H8···C19	2.7500
C14···C12	3.586 (3)	H8···Cl1^vii^	2.8400
C14···C16^vi^	3.562 (3)	H10···C18^ix^	3.0400
C15···C17^vi^	3.566 (3)	H12···Cl1	2.6600
C15···C12^i^	3.512 (3)	H12···C15^ii^	3.0900
C16···Cl1^i^	3.556 (2)	H12···C1	2.8500
C16···C14^vi^	3.562 (3)	H14···C12	2.9000
C17···C15^vi^	3.566 (3)	H14···C7	2.8100
C18···C20	3.312 (3)	H16···Cl1^i^	2.8500
C19···Cl1^vii^	3.3830 (19)	H17···N1^x^	2.6100
C19···Cl1^ii^	3.3076 (17)	H18···N1	2.9100
C20···C6	3.353 (3)	H18···C20	2.5900
C20···C18	3.312 (3)	H18···C19	2.9000
C1···H12	2.8500	H19A···Cl1^ii^	2.3400
C7···H14	2.8100	H19A···H6	2.5000
C8···H19A	3.0300	H19A···C8	3.0300
C8···H19B	3.0400	H19B···C8	3.0400
C12···H14	2.9000	H19B···H8	2.4100
C13···H2	2.7500	H19B···Cl1^vii^	2.4600
			
C1—P1—C7	111.03 (8)	C2—C3—H3	120.00
C1—P1—C13	109.26 (8)	C4—C3—H3	120.00
C1—P1—C19	108.56 (8)	C3—C4—H4	120.00
C7—P1—C13	110.71 (8)	C5—C4—H4	120.00
C7—P1—C19	106.81 (8)	C4—C5—H5	120.00
C13—P1—C19	110.43 (8)	C6—C5—H5	120.00
P1—C1—C2	120.70 (14)	C1—C6—H6	120.00
P1—C1—C6	119.17 (14)	C5—C6—H6	120.00
C2—C1—C6	120.13 (17)	C7—C8—H8	120.00
C1—C2—C3	119.6 (2)	C9—C8—H8	120.00
C2—C3—C4	120.2 (3)	C8—C9—H9	120.00
C3—C4—C5	120.7 (2)	C10—C9—H9	120.00
C4—C5—C6	120.0 (2)	C9—C10—H10	120.00
C1—C6—C5	119.36 (19)	C11—C10—H10	120.00
P1—C7—C8	121.33 (14)	C10—C11—H11	120.00
P1—C7—C12	118.64 (14)	C12—C11—H11	120.00
C8—C7—C12	120.02 (17)	C7—C12—H12	120.00
C7—C8—C9	119.62 (18)	C11—C12—H12	120.00
C8—C9—C10	120.1 (2)	C13—C14—H14	120.00
C9—C10—C11	120.5 (2)	C15—C14—H14	120.00
C10—C11—C12	120.3 (2)	C14—C15—H15	120.00
C7—C12—C11	119.5 (2)	C16—C15—H15	120.00
P1—C13—C14	120.47 (14)	C15—C16—H16	120.00
P1—C13—C18	120.37 (14)	C17—C16—H16	120.00
C14—C13—C18	119.13 (17)	C16—C17—H17	120.00
C13—C14—C15	120.1 (2)	C18—C17—H17	120.00
C14—C15—C16	120.1 (2)	C13—C18—H18	120.00
C15—C16—C17	120.4 (2)	C17—C18—H18	120.00
C16—C17—C18	120.4 (2)	P1—C19—H19A	109.00
C13—C18—C17	119.83 (19)	P1—C19—H19B	109.00
P1—C19—C20	114.38 (13)	C20—C19—H19A	109.00
N1—C20—C19	178.6 (2)	C20—C19—H19B	109.00
C1—C2—H2	120.00	H19A—C19—H19B	108.00
C3—C2—H2	120.00		
			
C7—P1—C1—C2	100.11 (16)	P1—C1—C6—C5	−178.74 (15)
C7—P1—C1—C6	−79.73 (16)	C2—C1—C6—C5	1.4 (3)
C13—P1—C1—C2	−22.28 (17)	C1—C2—C3—C4	−0.6 (3)
C13—P1—C1—C6	157.89 (14)	C2—C3—C4—C5	1.5 (4)
C19—P1—C1—C2	−142.77 (15)	C3—C4—C5—C6	−0.9 (4)
C19—P1—C1—C6	37.40 (17)	C4—C5—C6—C1	−0.5 (3)
C1—P1—C7—C8	146.77 (15)	P1—C7—C8—C9	−179.84 (17)
C1—P1—C7—C12	−34.67 (18)	C12—C7—C8—C9	1.6 (3)
C13—P1—C7—C8	−91.68 (17)	P1—C7—C12—C11	−178.8 (2)
C13—P1—C7—C12	86.88 (17)	C8—C7—C12—C11	−0.3 (3)
C19—P1—C7—C8	28.58 (18)	C7—C8—C9—C10	−1.0 (4)
C19—P1—C7—C12	−152.86 (16)	C8—C9—C10—C11	−1.0 (4)
C1—P1—C13—C14	95.07 (17)	C9—C10—C11—C12	2.4 (5)
C1—P1—C13—C18	−82.80 (17)	C10—C11—C12—C7	−1.8 (4)
C7—P1—C13—C14	−27.51 (18)	P1—C13—C14—C15	−177.19 (17)
C7—P1—C13—C18	154.62 (15)	C18—C13—C14—C15	0.7 (3)
C19—P1—C13—C14	−145.59 (16)	P1—C13—C18—C17	177.16 (17)
C19—P1—C13—C18	36.54 (18)	C14—C13—C18—C17	−0.7 (3)
C1—P1—C19—C20	48.05 (15)	C13—C14—C15—C16	0.1 (3)
C7—P1—C19—C20	167.85 (13)	C14—C15—C16—C17	−0.9 (3)
C13—P1—C19—C20	−71.71 (15)	C15—C16—C17—C18	0.9 (3)
P1—C1—C2—C3	179.31 (16)	C16—C17—C18—C13	0.0 (3)
C6—C1—C2—C3	−0.9 (3)		

Symmetry codes: (i) −*x*+3/2, *y*−1/2, −*z*+1/2; (ii) −*x*+3/2, *y*+1/2, −*z*+1/2; (iii) *x*−1/2, −*y*+1/2, *z*−1/2; (iv) −*x*+3/2, *y*+1/2, −*z*+3/2; (v) −*x*+1, −*y*+1, −*z*+1; (vi) −*x*+2, −*y*, −*z*+1; (vii) *x*+1/2, −*y*+1/2, *z*+1/2; (viii) *x*−1/2, −*y*+1/2, *z*+1/2; (ix) *x*+1/2, −*y*+1/2, *z*−1/2; (x) −*x*+3/2, *y*−1/2, −*z*+3/2.

### Hydrogen-bond geometry (Å, °)

**Table:**

*D*—H···*A*	*D*—H	H···*A*	*D*···*A*	*D*—H···*A*
C12—H12···Cl1	0.93	2.66	3.479 (2)	147
C17—H17···N1^x^	0.93	2.61	3.530 (3)	171
C19—H19A···Cl1^ii^	0.97	2.34	3.3076 (17)	173
C19—H19B···Cl1^vii^	0.97	2.46	3.3830 (19)	160
C15—H15···Cg1^i^	0.93	3.06	3.890 (3)	150

Symmetry codes: (x) −*x*+3/2, *y*−1/2, −*z*+3/2; (ii) −*x*+3/2, *y*+1/2, −*z*+1/2; (vii) *x*+1/2, −*y*+1/2, *z*+1/2; (i) −*x*+3/2, *y*−1/2, −*z*+1/2.
